# Skeletal Muscle Heat Shock Protein Content and the Repeated Bout Effect

**DOI:** 10.3390/ijms25074017

**Published:** 2024-04-04

**Authors:** Marius Locke, Giovanni Bruccoleri

**Affiliations:** Faculty of Kinesiology and Physical Education, University of Toronto, 55 Harbord Street, Toronto, ON M5S 2W6, Canada; giovanni.bruccoleri@mail.utoronto.ca

**Keywords:** rodent, tibialis anterior, skeletal muscle damage, lengthening contractions, heat shock proteins

## Abstract

The “Repeated Bout Effect” (RBE) occurs when a skeletal muscle is preconditioned with a few lengthening contractions (LC) prior to exposing the muscle to a greater number of LC. The preconditioning (PC) results in significantly less damage and preservation of force. Since it takes only a few LC to increase muscle heat shock protein (HSP) content, it was of interest to examine the relationship between HSPs and the RBE. To do this, one tibialis anterior (TA) muscle from Sprague–Dawley rats (n = 5/group) was preconditioned with either 0, 5, or 15 lengthening contractions (LC) and exposed to a treatment of 60 LC 48 h later. Preconditioning TA muscles with 15 LC, but not 5 LC, significantly elevated muscle αB-crystallin (*p* < 0.05), HSP25 (*p* < 0.05), and HSP72 content (*p* < 0.001). These preconditioned TA muscles also showed a significantly (*p* < 0.05) reduced loss of active torque throughout the subsequent 60 LC. While there was a trend for all preconditioned muscles to maintain higher peak torque levels throughout the 60 LC, no significant differences were detected between the groups. Morphologically, preconditioned muscles appeared to show less discernible muscle fiber damage. In conclusion, an elevated skeletal muscle HSP content from preconditioning may contribute to the RBE.

## 1. Introduction

Skeletal muscle contraction is typically described as a concentric or shortening contraction (SC), whereby the contractile proteins, actin and myosin, slide past each other, resulting in the shortening of the sarcomere, the fiber, and the muscle. However, if the resistance/load imposed on a contracting muscle is greater than the force generated by the muscle, the myofilaments are stretched and pulled apart while under tension. Usually against gravity, these forcible contractions are commonly termed “eccentric contractions”, although a more appropriate term is a “lengthening contraction” (LC). It is well documented that a significant number of LC can result in muscle (fiber) damage, which is typically characterized by swollen and/or necrotic fibers [1,2], ruptured membranes [2,3,4], immune cell infiltration [2,3], and Z-disk streaming [1,4], and perhaps most importantly, a loss of force occurs [4,5,6,7]. While various reasons likely contribute to the damage, the velocity of contraction has been shown to play a role [8].

Skeletal muscle exhibits remarkable plasticity, and perhaps it is not surprising that the muscle damage and loss of force known to occur following an acute, yet severe bout of LC (~40–100) can be reduced if a less severe bout of LC (~1–20) is experienced prior to the more severe damaging bout. This protective response, which is termed the “Repeated Bout Effect” (RBE), has been observed in both animals and humans [5,6,9,10,11,12]. It should be noted that the initial, less severe “preconditioning” bout of LC is usually followed by a recovery period that is presumed to allow some sort of adaptation to occur, which would be likely responsible for the protection imparted by the preconditioning. Although the RBE has been known for many years [13], the exact mechanism(s) underlying this phenomenon remain unclear, although cellular, neural, and mechanical mechanisms have been proposed [13].

In the past, preconditioned LC bouts often resulted in a discernable amount of muscle damage; however, recent evidence has demonstrated that preconditioning muscles with a few LC, with no discernable muscle damage, can also cause an RBE [9,10]. This suggests that skeletal muscle repair/remodeling may not be a main factor and that other events may be more important. One possible explanation for the RBE that has received little attention is the expression of the protective proteins known as “heat shock” proteins (HSPs; [1,2,14,15,16,17,18]). Indeed, the overexpression of HSPs in both cardiac and skeletal muscles has been shown to provide protection against certain stressors [19,20]. For example, the transgenic of HSP72 in mouse heart has been shown to protect hearts from ischemic injury. HSP72 overexpression in aged skeletal muscles removed the 44% force deficit that was observed in muscles from non-transgenic aged mice [14]. The small HSPs HSP25 and alpha-B crystallin (αB-crystallin) have been demonstrated to have a relatively high expression in unstressed skeletal muscle fibers and are known to translocate to the Z-disk of sarcomeres where they may function as stabilizers [21,22,23]. In view of the proposed functions for these HSPs, it follows that an elevated HSP content may provide some protection to muscle fibers during LC. Previous work has shown that 5 LC result in no discernable damage and only a slight increase in muscle HSP content, while 15 LC result in a more robust HSP increase [1]. Thus, the purpose of the present study was to use 5 and 15 LC as preconditioning treatments to determine whether these protocols differ in their elevation of HSPs in skeletal muscle and confer an RBE. By understanding the relationship between skeletal muscle HSP content and muscle protection, methods on how to repair or enhance muscle protection might be developed for better recovery outcomes.

## 2. Results

At the time of euthanasia, the mean body mass was 422.2 ± 14.2 g. No significant differences were detected in body mass between the groups (Table 1). To normalize treatments, the TA muscle mass values are provided as a ratio of each animal’s stimulated TA muscle mass to its non-stimulated contra-lateral (CL) TA muscle mass. In general, ratios of close to 1.0 (0.95–1.04) were observed, suggesting that either no change in muscle mass occurred, or if any change did occur, it returned to the original mass by 48 h (Table 1). When statistically assessed, no significant differences in TA muscle mass ratios were detected between the groups. Thus, at 48 h after all LC protocols, the masses of the stimulated TA muscles were comparable to those of the CL TA muscles.

### 2.1. Preconditioning

**Figure 1:**
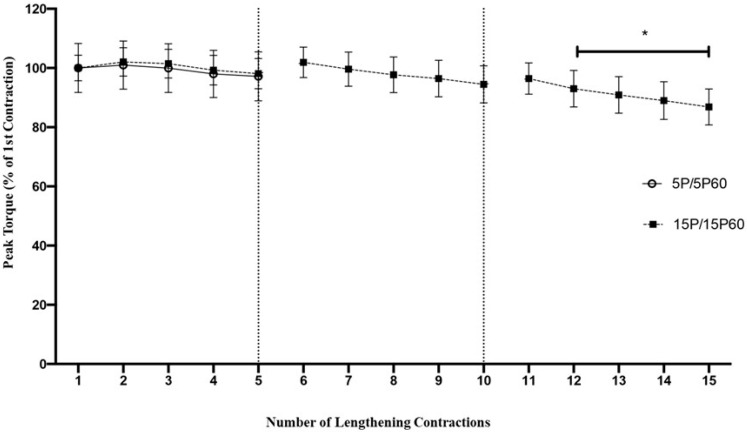
Fifteen LC but not five LC significantly decreased peak torque. Peak torque dropped approximately 3% following 5 LC but was reduced by approximately 13% following 15 LC. Torque was significantly reduced at the 12th LC and thereafter relative to the first contraction. * *p* < 0.001. n = 10/group. Values are expressed as mean ± SEM.

TA muscles preconditioned with 5 LC showed only a 2.8 ± 8.3% non-significant reduction in peak torque (Figure 1). Similarly, TA muscles subjected to 15 LC also showed a non-significant reduction (1.9 ± 5.1%) relative to the first contraction at the 5th LC. After the 5th LC, the peak torque showed no further reduction, such that a non-significant drop of 5.54 ± 6.30% was observed at the 10th LC. From the 12th contraction and thereafter, a significant (*p* < 0.001) loss of torque was detected, such that by the 15th LC, there was a significant 13.1 ± 6.1% reduction in torque. Thus, when compared to the first contraction, the peak torque was not significantly decreased with 5 preconditioned LC, while 15 preconditioned LC significantly reduced peak torque at the 12th contraction and thereafter.

### 2.2. Peak Torque Loss following 60 LC

**Figure 2:**
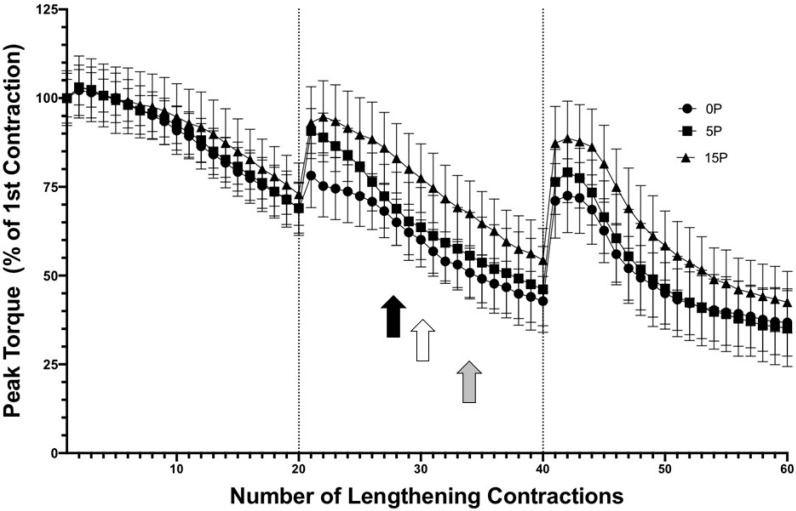
Neither 5 LC nor 15 LC demonstrated an RBE on peak torque. Three conditions (0P60, 5P60, and 15P60) underwent 3 sets of 20 LC with 5 min of rest between sets. By the end of the 60th LC, the peak torque had decreased by 63.17 ± 8.8%, 64.74 ± 9.44%, and 57.46 ± 6.48% in the 0P60, 5P60, and 15P60 groups, respectively. No significant (n.s.) differences between the groups were detected. The commencement of in-group differences in peak torque relative to the first contraction for 0P60, 5P60, and 15P60 are represented by the black, white, and gray arrows, respectively (*p* < 0.05). Values are expressed as mean ± SEM.

The analyses of the peak TA muscle torque expressed relative to the first contraction showed no significant differences between the groups (Figure 2), and no significant differences from the first contraction were detected during the first 20 repetitions (set 1) for all groups. However, when compared to the first contraction, the non-preconditioned muscles were significantly reduced at the 28th LC (black arrow in Figure 2) and remained significantly reduced until the 40th LC. Directly after the rest between sets 2 and 3, the peak torque recovered but was again significantly reduced between the 45th and 60th LC (*p* < 0.05). Similarly, the peak torque of the TA muscles preconditioned with 5 LC was significantly decreased from the 30th (white arrow in Figure 2) to the 40th LC and from the 46th to 60th LC (*p* < 0.05). Interestingly, the TA muscles preconditioned with 15 LC retained peak torque until the 34th LC (gray arrow in Figure 2), but thereafter showed a significant reduction in peak torque until the 40th LC, and again from the 48th to 60th LC (*p* < 0.05). This pattern of torque loss suggested that preconditioned TA muscles maintained higher peak torque values for a greater number of contractions when compared to the non-preconditioned muscles or muscles preconditioned with 5 LC. However, there were no significant differences between the groups.

When subjected to an LC, the peak muscle torque achieved can be divided into active and passive torque components. The active torque component reflects the maximal tetanic tension reached prior to the lever moving, while the passive (or lengthening) torque component consists of the resistance provided by the muscle while being forcibly lengthened.

**Figure 3:**
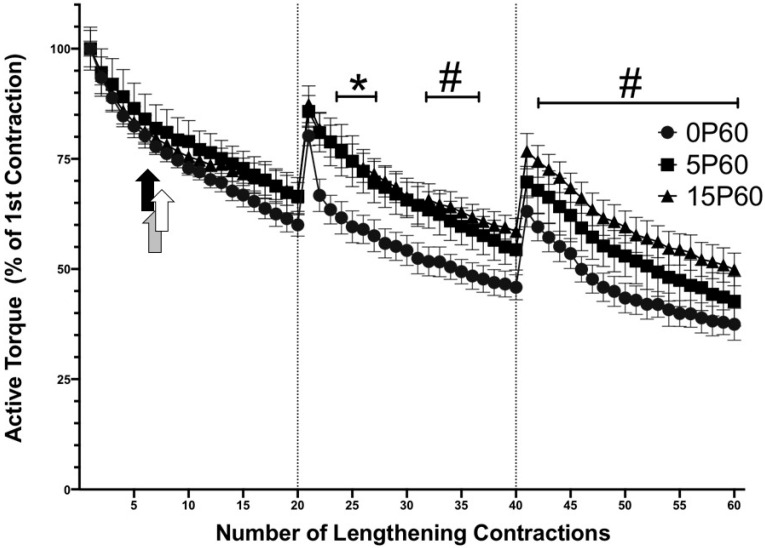
Preconditioning with 15 LC demonstrated an RBE on active torque. Both 5P60 and 15P60 sustained greater active torque for a few contractions in the second set, relative to 0P60 * *p* < 0.05. The 15P60 group retained greater active torque values throughout the second and third sets, relative to 0P60 # *p* < 0.05. The commencement of in-group drops in active torque relative to the first contraction are represented by the black, white, and gray arrows in the 0P60, 5P60, and 15P60 groups, respectively (*p* < 0.05). Values are expressed as mean ± SEM.

When the active torque component was assessed relative to the first contraction, a non-significant decline of 39.9 ± 2.6% in active torque was observed in TA muscles from animals with no preconditioning (0P60) after 20 LC. Similarly, there was a non-significant drop in active torque of 33.5 ± 4.2% in the TA muscles of the 5P60 group and 33.7 ± 3.3% in the TA muscles of the 15P60 group following the 20th LC (Figure 3). By the 20th LC, the active torque of skeletal muscles from the 15P60 group was ~6.3% higher than that of the 0P60 group but only ~0.2% higher than that of the 5P60 group.

In the second set, TA muscles from the preconditioned groups showed less of a drop in active torque than muscles from the 0P60 group. In contrast to peak torque, the active torque of the 5P60 group was maintained higher between the 22nd and 26th LC when compared to that of the 0P60 group (*p* < 0.05). However, by the 27th LC, the active torque declined and became similar to the active torque in the 0P60 group. Most importantly, the TA muscles from the 15P60 group demonstrated some active torque protection throughout the majority of the second set (22nd–29th and 32nd–37th LC) when compared to those of the 0P60 group (*p* < 0.05). By the 40th LC, the TA muscles from the 15P60 group generated active torque values that were ~12.7% and ~4.2% above the active torque generated by the TA muscles from the 0P60 and 5P60 groups, respectively. By the 40th LC, TA muscle active torque had declined by 54.1 ± 2.8% in 0P60, 45.6 ± 4.6% in 5P60, and 41.5 ± 2.9% in 15P60, and no statistical significances were observed between the groups.

In the final LC set (repetitions, 41–60), skeletal muscles subjected to preconditioning retained a higher active torque when compared to the non-preconditioned muscles (Figure 3). Between the 40th and 60th LC, the active torque of the TA muscles subjected to 15 LC was significantly different (*p* < 0.05) (higher) than the TA muscles not subjected to preconditioning. No statistical differences were observed between the TA muscles from the 15P60 and 5P60 groups, nor between the 5P60 and 0P60 groups. By the 60th LC, the animals preconditioned with 15 LC (15P60) showed a reduction of only 50.1 ± 3.7%, compared to a loss of 62.5 ± 3.6% and 57.3 ± 5.6% in the 0P60 and 5P60 groups, respectively (Figure 3). Thus, by the 60th LC, the muscles from the 15P60 group showed ~12.4% and ~7.2% higher active torque values compared to the muscles from the 0P60 and 5P60 groups, respectively.

### 2.3. HSP Content

#### 2.3.1. αB-Crystallin

**Figure 4:**
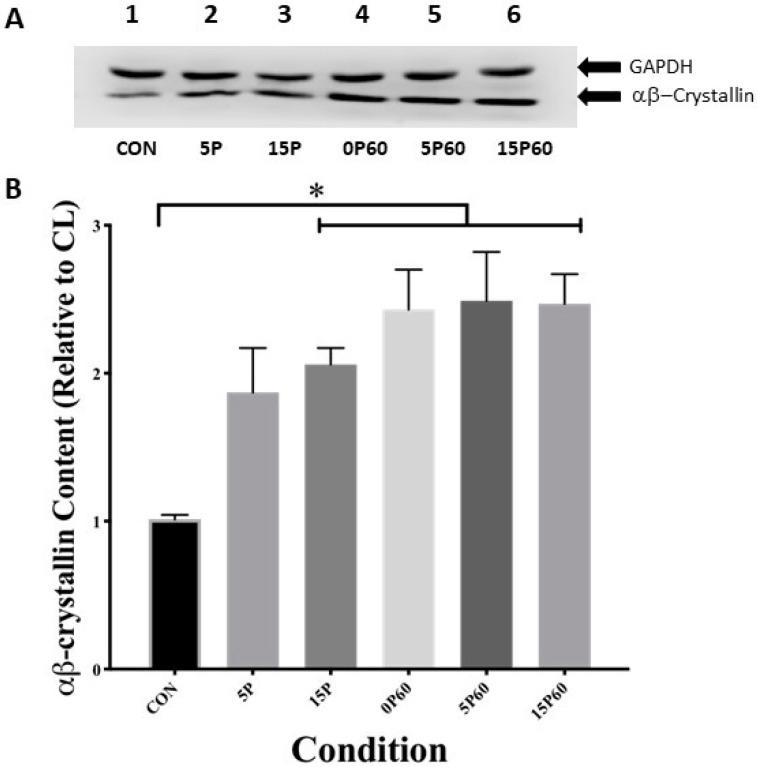
Preconditioning with 15 LC increased skeletal muscle αB-crystallin content similar to that of 60 LC. (**A**) Western blot analysis of αB-crystallin and GAPDH content for each condition. (**B**) The 15P, 0P60, 5P60, and 15P60 demonstrated elevated αB-crystallin content relative to CON * *p* < 0.05, whereas 5P had no effect on αB-crystallin content. Values are expressed as mean ± SEM.

To assess the TA muscle αB-crystallin (≈22 kDa) content, muscle homogenates were subjected to SDS-PAGE and Western blotting procedures as described in Methods and Materials. The values obtained from the densitometric scanning of the bands on the blots from each stimulated TA muscle are expressed relative to the αB-crystallin content in their respective CL TA, and the data expressed as a fold-increase. GAPDH (≈36 kDa) was used as a total protein loading control, and visual inspection indicated a similar total protein loading per lane (Figure 4A). As expected, the αB-crystallin content in the TA muscles from control animals was 1.0 ± 0.0. In the TA muscles following preconditioning with 5 LC, no change was observed in αB-crystallin content (Figure 4B) (1.9 ± 0.3). In contrast, the TA muscles preconditioned with 15 LC showed a significant (*p* < 0.05) increase in αB-crystallin content, such that a 2.06 ± 0.11-fold elevation relative to the muscles in the control group was detected. When subjected to 60 LC, the TA muscles with no preconditioning showed a 2.4 ± 0.3-fold increase in αB-crystallin content, which was similar to the TA muscles from the 5P60 (2.5 ± 0.3-fold) and 15P60 groups (2.5 ± 0.2-fold increase) (*p* < 0.05) and later subjected to 60 LC (Figure 4B). Despite these increases, there were no significant differences in TA muscle αB-crystallin content after 60 LC between preconditioned and non-preconditioned muscles.

#### 2.3.2. HSP25

**Figure 5:**
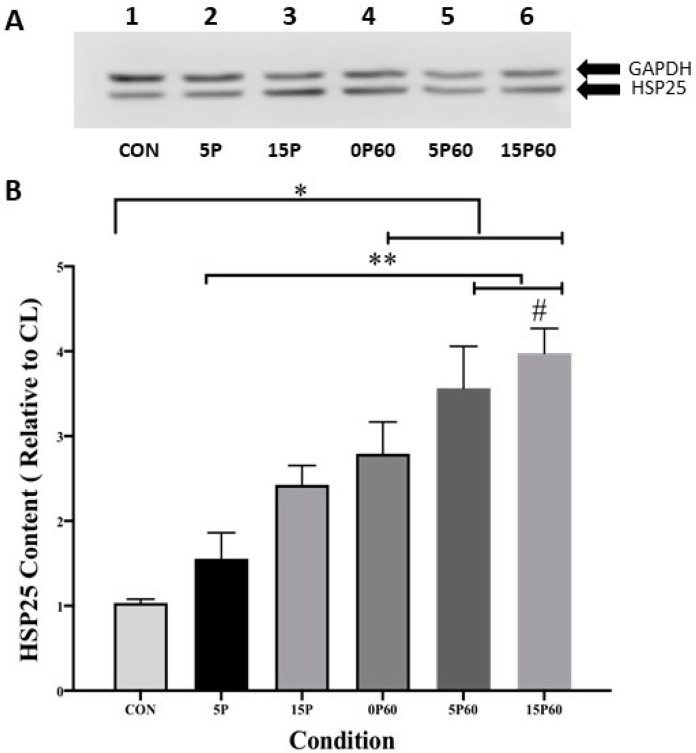
HSP25 content increased in proportion to the number of LC. (**A**) Western blot analysis of HSP25 and GAPDH content in each condition. (**B**) The 15P, 0P60, 5P60, and 15P60 all significantly elevated HSP25 content relative to CON * *p* < 0.05. Additionally, 5P60 and 15P60 significantly elevated HSP25 content compared to 5P ** *p* < 0.01 and 15P60 HSP25 content was significantly higher than that in 15P # *p* < 0.05. Values are expressed as mean ± SEM.

Similar to those for αβ-crystallin, data for HSP 25 are expressed as a ratio of the stimulated and CL TA muscle values and are expressed as a fold-increase (Figure 5A). As expected, HSP25 content in the control (unstressed) TA muscle remained unchanged (1.0 ± 0.0), while preconditioning with 5 LC insignificantly increased the skeletal muscle HSP25 content 1.6 ± 0.3-fold. TA muscles preconditioned with 15 LC showed a significant (*p* < 0.05) 2.6 ± 0.2-fold elevation of HSP25 content when compared to the control (unstressed) muscle. In all conditions subjected to 60 LC, HSP25 content was significantly (*p* < 0.01) elevated relative to the control (unstressed) muscle. Increases in HSP25 content of 2.8 ± 0.4-, 3.6 ± 0.5-, and 3.9 ± 0.3-fold were observed for the 0P60, 5P60, and 15P60 groups, respectively (Figure 5B). Taken together, these data suggest that muscle HSP25 increases following LC and that the presence of HSP25 in muscles prior to a second stress may not lessen any subsequent increases.

#### 2.3.3. HSP72

**Figure 6:**
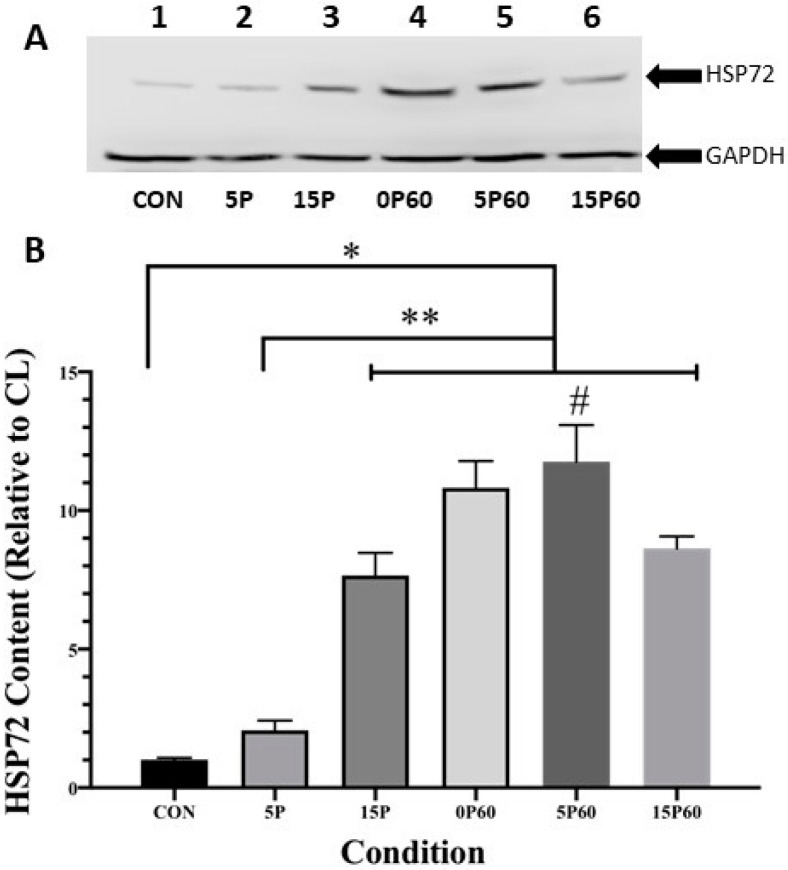
15 LC but not 5 LC elevated HSP72. (**A**) Western blot analysis of HSP72 and GAPDH content in the various conditions. (**B**) All conditions with the exception of 5P significantly elevated HSP72 content relative to CON * *p* < 0.0001. HSP72 content in 15P, 0P60, 5P60, and 15P60 was significantly higher than 5P ** *p* < 0.0001. Only 5P60 demonstrated HSP72 content greater than 15P # *p* < 0.05. Values are expressed as mean ± SEM.

Unlike the sHSPs, which tend to exhibit relatively high constitutive levels of expression in unstressed muscle [16,21], the inducible isoform of the HSP70 family, HSP72, showed a relatively low basal expression in the unstressed (control) muscle (Figure 6A—lane 1). When expressed as a ratio of CL TA to LC TA content, HSP72 content in the TA muscles from the unstressed (control) muscle was, as expected, similar (1.0 ± 0.1-fold). The HSP72 content in the TA muscles from the 5P group showed a non-significant (2.1 ± 0.4-fold) increase. In contrast, when TA muscles were preconditioned with 15 LC, HSP72 content was significantly elevated 7.7 ± 0.8-fold higher than the controls (*p* < 0.0001). This was also significantly different from the TA muscles subjected to 5 LC (*p* < 0.0001). Thus, preconditioning with 15 LC, but not 5 LC, significantly elevated HSP72 content.

When all groups (0, 5, and 15 LC) were subjected to 60 LC, and the TA muscle HSP72 content was compared to the unstressed (control) TA muscle, the HSP72 content was significantly elevated, such that increases of 10.8 ± 0.9- and 11.8 ± 1.3-fold were observed for 0 or 5 preconditioned LC, respectively. Interestingly, although TA muscles preconditioned with 15 LC followed by 60 LC also showed a significant (*p* < 0.0001) 8.6 ± 0.4-fold elevation in HSP 72 content, it was considerably lower than the other conditions (Figure 6B). Thus, when exposed to a subsequent set of LC, the prior treatment with 15 LC but not 5 LC appeared to dampen the HSP72 response.

### 2.4. Morphological Properties

#### 2.4.1. Fiber Morphology

**Figure 7:**
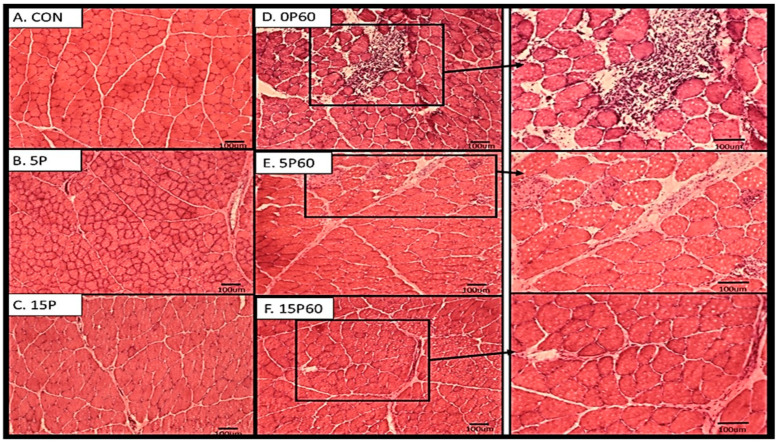
Preconditioning treatments showed morphological protection from 60 LC. Upon visual inspection, the CON (**A**), 5P (**B**), and 15P (**C**) appeared to have no notable change in morphology with the exception of immune cell infiltration as the number of LC increased. Following 60 LC, 0P60 (**D**) demonstrated the highest evidence of morphological damage, whereas 5P60 (**E**) and 15P60 (**F**) each showed less evidence of damage, respectively. Scale indicates 100 μm.

Frozen cross-sections (10–20 μm) from TA muscles were stained using the hematoxylin and eosin protocol as described in Methods and Materials. Visual assessment showed TA muscles from unstressed or contra-lateral muscles showed normal polygonal-shaped muscle fibers, with intact morphological features and no evidence of immune cell infiltration or rounding (Figure 7A). TA muscles subjected to 5 LC (5P) also demonstrated no notable changes in morphology, with only slight evidence of immune cell infiltration in a few localized areas (Figure 7B). TA muscles that underwent 15 LC (15P) also showed a similarly slight level of muscle fiber damage and fiber rounding when compared to the TA muscles from the 5P or CON groups (Figure 7C).

Visual inspection of the TA muscles subjected to 60 LC with no preconditioning (0P60) demonstrated substantial amounts of muscle fiber damage, including increased necrotic fibers, swollen fibers, as well as immune cell infiltration (Figure 7D). The muscle fibers from the 5P60 group also showed some evidence of fiber damage as indicated by swollen fibers, centralized nuclei, and immune cell infiltration (Figure 7E). However, the damage observed was not as extensive as the damage observed in the TA from the control group. When TA muscle fibers from the 15P60 group were examined, there were a few localized areas of muscle damage observed. While not quantified, visually, it appeared that there was a trend of less muscle fiber damage with preconditioning treatments.

#### 2.4.2. Electron Microscopy

**Figure 8:**
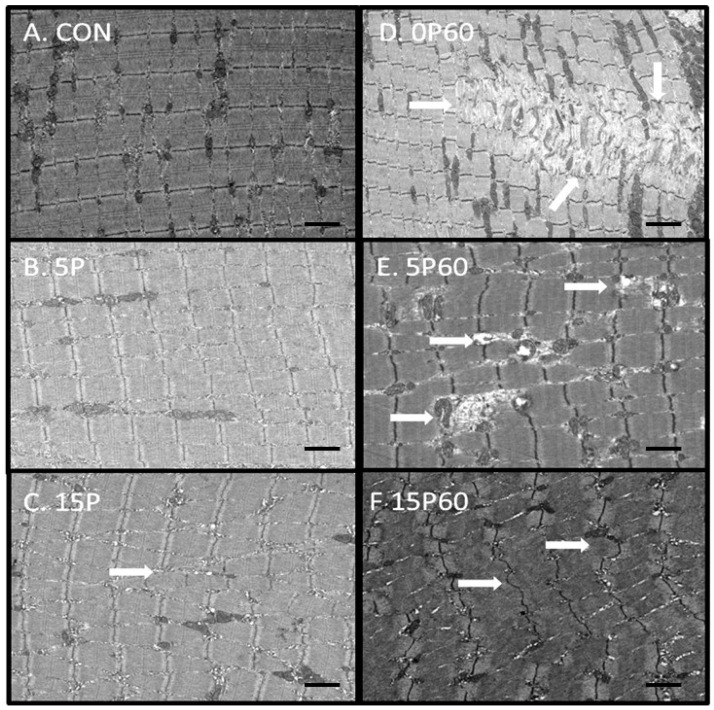
Both preconditioning treatments appeared to reduce morphological disruptions in the sarcomeres. Upon visual inspection, the CON (**A**) and 5P (**B**) appeared to have no notable change in sarcomere organization or changes to integrity. The 15P (**C**) appeared to provide some evidence for Z-disk streaming. Following 60 LC, the 0P60 (**D**) demonstrated the greatest evidence of sarcomeric disintegration and discontinuity, whereas the 5P60 (**E**) and 15P60 (**F**) each showed localized evidence of damage indicated by the white arrows. Scale indicates 2 μm.

To further assess morphological changes in the muscle fibers, TA muscles from each group were portioned (2–3 mm^2^) and prepared for EM as described in Methods and Materials. As expected, the unstressed TA muscles from the control group showed intact muscle fibers with normal sarcomere integrity and organization (Figure 8A). The TA muscles from the 5P group also appeared to have no notable disruptions or damage to the sarcomere (Figure 8B). In contrast, the TA muscles treated with 15 LC (15P) showed a few slight disruptions of sarcomere alignment and Z-disk streaming (Figure 8C). When the TA muscles subjected to 60 LC were examined, the TA muscles with no preconditioned LC showed a significant amount of damage as indicated by the relatively large areas within muscle fibers where the sarcomere integrity appeared discontinuous (Figure 8D). A prior preconditioning with 5 and 15 LC appeared to reduce the extent of discernable damage and integrity of muscle fibers. Less evidence of muscle fiber damage was observed in the 5P60 group relative to the 0P60 group; although, there was some evidence of Z-disk streaming and sarcomere disruption (Figure 8E). Of the three groups subjected to 60 LC, the muscle fibers from the 15P60 group showed the least amount of disruption and continuity in sarcomere integrity. However, it should be noted that the TA muscles from the 15P60 group did show some Z-disk streaming (Figure 8F). Overall, it appeared that preconditioning with 15 LC resulted in the least amount of muscle fiber damage at the ultrastructural level.

## 3. Discussion

Exercise requires skeletal muscles to generate tension by one of three contraction types: isometric, shortening, or lengthening. Unless excessive, isometric and shortening contractions tend not to cause muscle damage [1,7]. In contrast, when a muscle is lengthened while under tension, such as with LC, it often results in muscle damage and a subsequent loss of force [1,2,12,13]. Skeletal muscle is inherently plastic and capable of adapting to the demands of the exercise; however, it generally takes several sessions before any improvements are detected. This is in contrast to the RBE phenomenon, where a muscle is preconditioned using only a single exercise bout consisting of only a few (~1–20) LC, prior to a (delayed) second bout that usually consists of a greater (~ 50 or more) number of damaging LC. The consistently observed result is that skeletal muscles perform better when provided with PC than without [9,10]. The aim of the present study was to compare two different PC protocols and assess the relationship between muscle HSP content and the RBE. The novel finding from the present study was that skeletal muscles preconditioned with 15, but not 5 LC, showed an elevated muscle HSP content and that the PC-associated increase in HSP content was associated with less active torque loss when subjected to 60 LC. While the relationship between HSPs and the RBE is largely correlative and requires further experimentation, the present study adds to the mounting evidence that suggests that HSPs protect skeletal muscle during episodes of stress [15,16,19,20,21]. In addition, it also implicates HSPs as possibly contributing to the RBE.

In the present study, TA muscles were assessed for contractile, morphological, and biochemical properties after PC with 0, 5, or 15 LC, prior to being subjected to 60 LC. Although no significant differences in peak or passive torque were observed between the groups (PC vs. no PC), there was a significant difference in active torque detected. PC TA muscles with 15 LC prior to being subjected to 60 LC resulted in significantly less torque loss when compared to no PC, thereby confirming an RBE. In addition to the RBE observed for active torque between the groups, there were also trends that supported an RBE. For example, throughout the entire 60 LC, the magnitude of peak torque loss was always greatest for the non-PC group, followed by the 5 LC PC group, and finally by the 15 LC PC group. This pattern of peak torque drop was consistent with an RBE. In addition, the exact repetition where a significant drop in peak force was detected was also related to the PC treatment. For example, when compared to the first contraction, the pattern of torque loss was such that during the 60 LC, a significant loss in muscle peak torque was detected at the 28th, 30th and 34th repetitions when preconditioned with 0, 5, or 15 LC, respectively. Thus, when compared to muscles with no preconditioning or with only 5 LC, the muscles preconditioned with 15 LC tended to perform better and were capable of sustaining higher torque values for a greater number of LC before showing a significant loss of torque. The pattern of torque loss tended to indicate that the greater the PC, the later the onset of torque loss. Furthermore, when muscle morphology was assessed by light and electron microscopy, the extent of muscle damage (after 60 LC) showed less muscle damage observed with PC.

While there are likely many factors involved with this observed RBE reported herein, it is tempting to speculate that HSPs may play a key role. HSPs are rapidly synthesized in all cells and tissues in response to a variety of stressors [14,16,24], including LC [1,2,4,7,14,15,20,21,22]. There are a number of interesting associations between HSPs, LC, and the RBE. First, one of the main intracellular targets reported to be damaged by LC is the Z-band [1,4]. While the exact reason(s) for this observation remain unclear, it may reflect a weak point in sarcomere integrity [15,21]. Interestingly, HSP25 and αB-crystallin are both known to be expressed at relatively high levels in skeletal muscle and located at the Z-band [15,20,21]. HSP25 has also been shown to translocate and bind to the Z-band [15,21]. More recently, Himori et al. [25] showed marked increases in both muscle αB-crystallin content and its steady-state binding to myofibrillar proteins in plantar flexor muscles of the adjuvant-induced arthritis of rats after LC. In the present study, the muscle content of HSP25, HSP72, and αB-crystallin were increased following 15 LC. Thus, it remains conceivable that one or more of these proteins may contribute to protecting muscle structures and thereby allow muscle fibers to cope with the challenge of 60 LC.

In addition to HSP25 and αB-crystallin, HSP72, the inducible member of the HSP70 family, has also been shown to protect skeletal muscle function [14,19]. In most cells and tissues, HSP72 is absent or present at very low levels. However, following the imposition of a protein damaging stressor such as LC, HSP72 rapidly increases. Interestingly, HSP72 has been shown to be expressed in a fiber-type-specific manner, such that Type I fibers express relatively high levels of HSP72, while Type II fibers express little, if any, HSP72 [17]. Since Type II fibers are more susceptible to LC damage than Type I fibers [26], it follows that any muscle fibers with an elevated HSP72 content would be better protected against the stress of LC. Thus, the more elevated TA muscle HSP72 content observed in the 15 LC preconditioning treatment reported herein might have been sufficient to allow fibers to cope with the stress of PC and also subsequently to provide protection when stressed with the 60 LC. In view of this, it may be the case that preconditioning with 15 LC, but not 5 LC, sets in motion an elevation of HSP expression to a level such that the more severe stress of 60 LC becomes a lesser relative stress. As a result, more fibers may be capable of withstanding the stress of the 60 LC bout and allow muscle force to be maintained. Regardless of the exact mechanism, the present study, coupled with previous work [11,14,25], supports the idea that HSPs provide protection to muscles from the stress of damaging skeletal muscle contractions.

This study had several limitations. First, the conclusions implicating the role of HSPs in protection and/or the RBE are largely correlative. More precise evaluations of specific HSPs using transgenic/knockout models may allow for more direct inferences. Second, while the sample size used was sufficient to detect significance for some of the measured parameters, it might be considered low for other parameters. Increasing the sample size may allow for a better detection for the peak and passive torque measurements, thereby resolving differences between trends and significance. A third limitation was that, although HSPs were shown to be elevated by Western blotting methods, this measurement reflected whole muscle values and may not have reflected localized areas within the muscle. It remains unknown if the observed elevated HSP expression was localized to the specific stressed areas, such as damaged myofibers, or whether the HSP levels were homogenous throughout the muscle. Lastly, the morphological data, while supportive, were largely subjective observations, and a stronger case would have to be made from some quantification.

## 4. Methods and Materials

**Figure 9:**
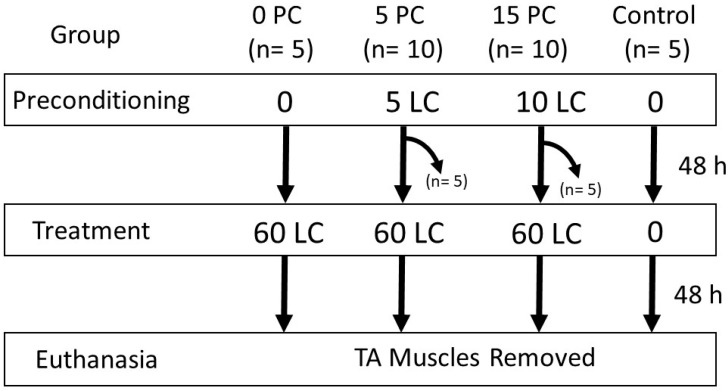
Study design. Preconditioning bouts of 0, 5, or 15 LC were followed forty-eight hours later and underwent 60 LC.

Thirty male Sprague–Dawley rats (387.1 to 491.1 g) were obtained from Charles River Laboratories (Montreal, QC, Canada), housed 2/cage, provided food and water *ad libitum*, and were maintained on a constant 12 h light/dark cycle. Experimental procedures were approved by the Animal Care Committee at the University of Toronto and were in accordance with Guidelines for the Canadian Council on Animal Care (July, 2023:AUP #20012987). Rats were divided into 4 main groups (Figure 9): a control group (n = 5) that received no LC, a group (n = 5) with no preconditioning and subjected to 60 LC (0P60), a group (n = 10) treated with 5 preconditioning LC prior to 60 LC (5P60), and a group (n = 10) treated with 15 preconditioning LC prior to 60 LC (15P60). To examine the muscle HSP content following preconditioning, five of the ten animals in each of the preconditioning groups (5 and 15 LC) were euthanized 48 h following PC and were not treated with 60 LC. Forty-eight hours after the last contraction, all animals were euthanized, and the TA muscles were removed and processed for HSP content and morphological assessment. A schematic outlining of the animals and procedures is shown in Figure 9.

### 4.1. Stimulation Protocol and Torque Generation

The stimulation protocol used was as previously described [1,2,4,7]. Animals were anesthetized using isoflurane and placed in a supine position on a 37 °C platform (806D, Aurora Scientific Inc., Aurora, ON, Canada) while an anesthesia of isoflurane/oxygen mixture was provided. The knee of the limb to be stimulated was secured between metal posts by the insertion of a needle (25 G × 1.5 inch), placed through the patellar tendon. The foot was secured to a foot-pedal, and two stimulating electrodes were subcutaneously placed above the TA muscle. Prior to LC, the maximal tetanic tension (MTT) for each TA muscle was applied as previously described [4,7]. In most cases, voltages of 6–12 V and 150 Hz were required. Muscle torque (g-cm) was recorded on a computer hardware interface (604A, Aurora Scientific Inc.) and analyzed through Dynamic Muscle Analysis Software version 5.01 (Aurora Scientific Inc.).

For the LC, each TA muscle was isometrically stimulated for 0.2 s, such that the MTT was attained. During an additional stimulation of 0.2 s, the muscle was lengthened throughout 38° of motion at an angular velocity of 127°/s. Following each muscle stimulation/contraction, the lever passively returned the limb to the starting position. Preconditioning consisted of either one or three sets of 5 LC with 1 min of rest provided between sets. The second (damaging) bout consisted of 60 LC divided into 3 sets of 20 LC with 5 min of rest between sets. Muscle torque was recorded on a computer hardware interface (604A, Aurora Scientific Inc.) and later analyzed using Dynamic Muscle Analysis Software (611A, Aurora Scientific Inc.).

Since muscle torque is proportional to muscle mass and for clarity of presentation, all muscle torque values are expressed as the percentage of the first contraction. Muscle torque was generated by the electrically stimulated TA muscles (measured in g·cm). Peak torque was calculated as the highest torque change measured during the entire contraction, while active torque was calculated as the highest torque at 0.2 s. Passive torque was determined by measuring the difference between peak torque and active torque.

### 4.2. Tissue Collection

The animals were anesthetized as described previously [2,4,7] and euthanized via cardiac exsanguination 48 h following either 5 or 15 LC preconditioning (n = 5) bouts or following the treatment with 60 LC. TA muscles from both left and right limbs were removed, weighed, and further divided into portions for biochemical and morphological analyses. The mid-belly portion of each TA muscle was embedded with an OCT compound and immersed in isopentane cooled in liquid nitrogen, while muscle portions for biochemical analysis were snap frozen in liquid nitrogen. All samples were stored at −70 °C until processed.

### 4.3. Biochemical Analysis

#### 4.3.1. Tissue Homogenization and Protein Determination

Frozen samples of the TA muscles (≈50–100 mg) were homogenized in 10-times radioimmunoprecipitation assay (RIPA) buffer (Sigma Aldrich, Saint Louis, MO, USA, #R0278) on ice with 1:100 of a protease inhibitor cocktail (Sigma Aldrich, #P8340) using a polytron grinder (IKA Labortechnik, Staufen im Breisgau, Germany). Muscle homogenates were isolated and the protein concentration determination was conducted using the method described by [27] using bovine serum albumin (BSA) for a standard.

#### 4.3.2. Sodium Dodecyl Sulfate Polyacrylamide Gel Electrophoresis and Western Blotting

Equal amounts of muscle protein homogenates (100–200 μg) were loaded in a 3% acrylamide stacking gel over a gradient of 5–15% acrylamide separating gel and subjected to sodium dodecyl sulfate polyacrylamide gel electrophoresis (SDS-PAGE), as previously described [1,2] using a Bio-Rad mini-protein II gel system. Following electrophoretic separation, proteins were transferred to nitrocellulose membranes (0.22 m pore size, Bio-Rad, Mississauga, ON, Canada) based on the technique described by [28] with the modifications described by [7]. In brief, following the SDS-PAGE separation of proteins, the transfer was performed at 50 v for 3 h with the ice pack changed at 1.5. Following transfer, nitrocellulose membranes were placed in a blocking solution consisting of 5% non-fat skim milk powder (NFSM) in Tris Buffered Saline (TBS:500 mmol/L NaCl, 20 mmol/L, Tris, pH 7.5) for 3 h. Membranes were washed twice in TBS and tween-20 (TTBS) for 5 min and incubated with a 1:1000 dilution of antibody for αB-crystallin (ADI-SPA-222, ENZO, Farmingdale, NY, USA), HSP25 (ADI-SPP-715, ENZO), or HSP72 (ADI-SPA-812, ENZO) 2% NFSM in TTBS for 4 h. A 1:5000 HRP labelled antibody for glyceraldehyde 3-phosphate dehydrogenase (GAPDH; Abcam, Cambridge, UK: 181602) was also added. The secondary antibodies for HSP25 and HSP72 consisted of a goat anti-rabbit IgG HRP-linked antibody (Cell Signaling, Danvers, MA, USA, #7074S). After a final 30 min wash in TBS, 4 mL of Luminata™ Forte Western HRP Substrate (Millipore Corporation, Billerica, MA, USA, #WBLUF0100) was added for 5 min in reduced light. Membranes were placed facedown and scanned using a Li-Cor C-Digit IS version 3.1 (Mandel Scientific, Guelph, ON, Canada, Model # CDG-001073 3600) light detection system at a high sensitivity setting for 12 min. Band densities were quantified using Image Studio software (Image Studio Digits, version 5.2.5), and protein bands (pixels) were quantified. Where appropriate, values are expressed relative to the unstressed CL limb.

### 4.4. Morphology

#### 4.4.1. Muscle Cross-Sectioning and Hematoxylin and Eosin Staining

The method for the assessment of morphology followed has been previously described by [1,2,4,7]. Mid-belly portions of the TA muscle tissues were sectioned (10–20 μm) in a transverse direction using an American Optical cryostat at −25 °C. Cut sections were placed on glass microscope slides, dried, and stored at −20 °C. The TA muscle cross-sections were subjected to hematoxylin and eosin (H and E) staining. Sections on slides were placed in Phosphate-buffered Saline (PBS) for 2 min and hematoxylin (HHS32, Sigma Aldrich, Saint Louis, MO, USA) for 5 min. The slides were rinsed in PBS twice for 2 min each prior to being placed in eosin (HT110180, Sigma) for 5 min. The slides were placed in 30%, 50%, 70%, 90%, 95%, and 100% ethanol for 2 min each prior to being twice subjected to a xylene substitute (A5597, Sigma) for 4 and 2 min, respectively. The cross-sections were sealed using a cytoseal (8311-4; Thermo Scientific, Waltham, MA, USA) and covered with coverslips. The slides were viewed under a Zeiss Axioskop light microscope (North York, ON, Canada) and photographed at 5× or 10× magnification using a Canon EOS Rebel 70D digital camera (Mississauga, ON, Canada) attached to the microscope using a Zarf Enterprise Microscope Adaptor (Spokane, Washington, DC, USA).

#### 4.4.2. Ultrastructure 

The procedure for EM was performed as previously described in previous studies [1,4]. Portions of the TA muscles were minced into pieces (2–3 mm^2^) and incubated in 3% glutaraldehyde in phosphate buffer (pH 7.4) at 4 °C. The sections were washed 3 times at 10 min, each using 0.1 mol/L Sorenson’s phosphate buffer and placed in 1% OsO_4_ for 1 h. The samples were washed again (3 times for 10 min each), and the pieces were placed in a series of solutions (50% ethanol for 10 min, 70% ethanol for 10 min, 80% ethanol 15 min, twice in 90% ethanol for 10 min, and twice in 100% ethanol for 10 min). The dehydrated pieces were then incubated in a series of ethanol: resin mixtures at 3:1 for 30 min, 1:1 mixture for 1 h, 1:3 mixture for an hour, and 100% Spurr’s resin overnight. Twenty-four hours later, samples were placed in fresh Spurr’s resin for six hours and embedded overnight at 65 °C. The muscle samples were sectioned, stained (3% uranyl acetate and Reynolds’ Lead Citrate) and examined (Hitachi HT7700 Transmission Electron Microscope, Tokyo, Japan). Grids were examined with a Hitachi HT7700 Transmission Electron Microscope (TEM) at the University of Toronto in the imaging facility of the Department of Cell and Systems Biology. Visual assessment of damage included Z-line streaming and/or sarcomere disarray.

### 4.5. Statistical Analysis

A two-way repeated measure ANOVA was used to determine statistical differences in loss of torque between the groups at the various time points. One-way ANOVAs were used to analyze HSP content. Statistical differences between the groups were further analyzed using Tukey’s post hoc analysis. Significance was set at an alpha of 0.05 (*p* < 0.05) and the findings are described as mean ± standard error of the mean (SEM). Prism Software version 6.0 was used to determine the statistical analysis and for figure generation.

## 5. Conclusions

The present study adds to the growing literature investigating the relationships between HSPs and muscle protection. Rodent TA muscles subjected to PC consisting of 15 LC showed less loss of active torque when compared to those with no PC, or with only 5 LC, thereby demonstrating an RBE. Given that these same PC muscles also showed an elevated HSP content, it further suggested HSPs may have played a protective role when subsequently challenged with a greater, more damaging bout of LC. Since various types of exercise have been shown to elevate muscle HSP content in both humans [6,9] and animals [11,12], it follows that the elevated expression of HSPs with training may play a key role in the benefits provided by exercise.

## Figures and Tables

**Table 1:** Body mass and relative TA/CL-TA mass. Body mass and ratio of the stimulated TA to contra-lateral TA (control). Values are expressed as mean ± SEM. n = 5 per group. No significant differences were detected.

Condition	Body Mass (g)	Stimulated TA/CL
Control	419.31 ± 16.89	0.95 ± 0.03
5 PCs	429.36 ± 17.94	0.98 ± 0.02
15 PCs	430.39 ± 9.94	0.98 ± 0.02
5 PCs–60 LC	411.08 ± 16.68	1.02 ± 0.02
0 PCs–60 LC	432.70 ± 15.42	1.04 ± 0.03
15 PCs–60 LC	410.59 ± 12.02	1.01 ± 0.02

## Data Availability

Data are contained within the article.

## References

[1] Bonello J.-P., Locke M. (2019). HSP72 expression is specific to skeletal muscle contraction type. Cell Stress Chaperones.

[2] Locke M., Salerno S.A. (2020). Ovariectomy alters lengthening contraction induced heat shock protein expression. Appl. Physiol. Nutr. Metab..

[3] Sidky S.R., Ingalls C.P., Lowe D.A., Baumann C.W. (2022). Membrane Proteins Increase with the Repeated Bout Effect. Med. Sci. Sports Exerc..

[4] Pollock-Tahiri E., Locke M. (2017). The cellular stress response of rat skeletal muscle following lengthening contractions. Appl. Physiol. Nutr. Metab..

[5] Hoppeler H., Herzog W. (2014). Eccentric Exercise: Many questions unanswered. J. Appl. Physiol..

[6] Hoppeler H. (2016). Moderate Load Eccentric Exercise; A Distinct Novel Training Modality. Front. Physiol..

[7] Holwerda A.M., Locke M. (2014). Hsp25 and Hsp72 content in rat skeletal muscle following controlled shortening and lengthening contractions. Appl. Physiol. Nutr. Metab..

[8] Ueda H., Tsuchiya Y., Ochi E. (2020). Fast-Velocity Eccentric Cycling Exercise Causes Greater Muscle Damage Than Slow Eccentric Cycling. Front. Physiol..

[9] Chen T.C., Lin M.-J., Lai J.-H., Chen H.-L., Yu H.-I., Nosaka K. (2018). Low-intensity elbow flexion eccentric contractions attenuate maximal eccentric exercise-induced muscle damage of the contralateral arm. J. Sci. Med. Sport.

[10] Lavender A.P., Nosaka K. (2008). Changes in markers of muscle damage of middle-aged and young men following eccentric exercise of the elbow flexors. J. Sci. Med. Sport.

[11] DiPasquale D.M., Bloch R.J., Lovering R.M. (2011). Determinants of the Repeated-Bout Effect After Lengthening Contractions. Am. J. Phys. Med. Rehabil..

[12] Lovering R.M., Roche J.A., Bloch R.J., De Deyne P.G. (2007). Recovery of Function in Skeletal Muscle Following 2 Different Contraction-Induced Injuries. Arch. Phys. Med. Rehabil..

[13] Hyldahl R.D., Chen T.C., Nosaka K. (2017). Mechanisms and Mediators of the Skeletal Muscle Repeated Bout Effect. Exerc. Sport Sci. Rev..

[14] McArdle A., Dillmann W.H., Mestril R., Faulkner J.A., Jackson M.J. (2004). Overexpression of HSP70 in mouse skeletal muscle protects against muscle damage and age-related muscle dysfunction. FASEB J..

[15] Frankenberg N.T., Lamb G.D., Overgaard K., Murphy R.M., Vissing K. (2014). Small heat shock proteins translocate to the cytoskeleton in human skeletal muscle following eccentric exercise independently of phosphorylation. J. Appl. Physiol..

[16] Huey K.A., Hilliard C.A., Hunt C.R. (2013). Effect of HSP25 loss on muscle contractile function and running wheel activity in young and old mice. Front. Physiol..

[17] Tupling A.R., Bombardier E., Stewart R.D., Vigna C., Aqui A.E., Henstridge D.C., Febbraio M.A., Hargreaves M., Frankenberg N.T., Lamb G.D. (2007). Muscle fiber type-specific response of Hsp70 expression in human quadriceps following acute isometric exercise. J. Appl. Physiol..

[18] Madden L.A., Sandström M.E., Lovell R.J., McNaughton L. (2008). Inducible heat shock protein 70 and its role in preconditioning and exercise. Amino Acids.

[19] Tupling A.R., Gramolini A.O., Duhamel T.A., Kondo H., Asahi M., Tsuchiya S.C., Borrelli M.J., Lepock J.R., Otsu K., Hori M. (2004). HSP70 Binds to the Fast-twitch Skeletal Muscle Sarco(endo)plasmic Reticulum Ca^2+^-ATPase (SERCA1a) and Prevents Thermal Inactivation. J. Biol. Chem..

[20] Thompson H.S., Clarkson P.M., Scordilis S.P. (2002). The repeated bout effect and heat shock proteins: Intramuscular HSP27 and HSP70 expression following two bouts of eccentric exercise in humans. Acta Physiol. Scand..

[21] Vissing K., Bayer M.L., Overgaard K., Schjerling P., Raastad T. (2009). Heat shock protein translocation and expression response is attenuated in response to repeated eccentric exercise. Acta Physiol..

[22] Koh T.J., Escobedo J. (2004). Cytoskeletal disruption and small heat shock protein translocation immediately after lengthening con-tractions. Am. J. Physiol. Cell Physiol..

[23] Paulsen G., Vissing K., Kalhovde J.M., Ugelstad I., Bayer M.L., Kadi F., Schjerling P., Hallén J., Raastad T. (2007). Maximal eccentric exercise induces a rapid accumulation of small heat shock proteins on myofibrils and a delayed HSP70 response in humans. Am. J. Physiol. Regul. Integr. Comp. Physiol..

[24] Touchberry C.D., Gupte A.A., Bomhoff G.L., Graham Z.A., Geiger P.C., Gallagher P.M. (2012). Acute heat stress prior to downhill running may enhance skeletal muscle remodeling. Cell Stress Chaperones.

[25] Himori K., Tatebayashi D., Ashida Y., Yamada T. (2019). Eccentric training enhances the αB-crystallin binding to the myofibrils and prevents skeletal muscle weakness in adjuvant-induced arthritis rat. J. Appl. Physiol..

[26] Macaluso F., Isaacs A.W., Myburgh K.H. (2012). Preferential type II muscle fiber damage from plyometric exercise. J. Athl. Train.

[27] Lowry O., Rosebrough M.J., Farr Randall A.L. (1951). Protein Measurement with the Folin Phenol Regent. J. Biol. Chem..

[28] Towbin H., Staehelin T., Gordon J. (1979). Electrophoretic transfer of proteins from polyacrylamide gels to nitrocellulose sheets: Procedure and some applications. Proc. Natl. Acad. Sci. USA.

